# Resistance to S-Methoprene Correlates with Pyriproxyfen Resistance in Field-Collected *Culex pipiens*

**DOI:** 10.3390/insects17030241

**Published:** 2026-02-26

**Authors:** Kristina Lopez, Patrick Irwin, Lyric C. Bartholomay, Mark E. Clifton

**Affiliations:** 1 North Shore Mosquito Abatement District, Northfield, IL 60093, USA; klopez@nsmad.org; 2 Northwest Mosquito Abatement District, Wheeling, IL 60007, USA; pirwin@nwmadil.gov; 3 Department of Pathobiological Sciences, University of Wisconsin—Madison, Madison, WI 53706, USA; lyric.bartholomay@wisc.edu

**Keywords:** larvicide, vector, susceptibility, mosquito control, insect growth regulator

## Abstract

The increasing prevalence of insecticide resistance threatens the efficacy of Integrated Mosquito Management (IMM) programs, particularly in regions reliant on chemical control for vector-borne disease prevention. Cross-resistance between active ingredients severely complicates essential resistance management strategies like product rotation. The previous literature suggests that laboratory-induced S-methoprene-resistant *Culex* species may be somewhat cross-resistant to pyriproxyfen, another juvenile hormone analog. This is a critical concern in the Chicago, IL, USA metropolitan area, where pyriproxyfen is used against mosquitoes with reduced susceptibility to S-methoprene. To determine if S-methoprene-resistant *Culex pipiens* are cross-resistant to pyriproxyfen in nature, we assessed 31 field-collected populations with significant S-methoprene exposure but varying histories of pyriproxyfen use by dose–response bioassays. *Culex pipiens* from all 31 sites exhibited high resistance to S-methoprene (RR_50_ > 10), and 84% were at least moderately resistant to pyriproxyfen (RR_50_ > 5). Reduced susceptibility to pyriproxyfen was confirmed in pyriproxyfen-unexposed populations, demonstrating potential S-methoprene-mediated cross-resistance. The level of S-methoprene resistance and the level of pyriproxyfen exposure significantly correlated with the level of pyriproxyfen resistance. We report the first widespread, high-level pyriproxyfen resistance in any medically significant mosquito species, underscoring the critical need for routine resistance surveillance and the adoption of integrated resistance management tactics utilizing larvicides with distinct modes of action.

## Simple Summary

The widespread problem of insecticide resistance is making it harder to protect communities from mosquito-borne illnesses like West Nile virus (WNV), forcing mosquito control teams to constantly look for new solutions. This study focused on *Culex pipiens* mosquitoes, the main vector for WNV in the Chicago area, which have been subjected to the larvicide S-methoprene for many years. We found that all 31 mosquito populations from the Chicago area were highly resistant to S-methoprene. We reasoned that this widespread reduced susceptibility to S-methoprene could predispose mosquitoes to a related larvicide called pyriproxyfen, even in areas where pyriproxyfen had never been used. This phenomenon, known as cross-resistance, means that the strategies currently used to reduce resistance, like rotation between or combining these two products, may not be effective. Applying pyriproxyfen only makes the resistance problem worse, though operational outcomes are unknown. This is the first report of such high levels of resistance to pyriproxyfen being found in mosquitoes. Our findings are an urgent warning to mosquito control programs globally. It is important to monitor resistance more closely and adopt new methods that use different types of chemicals to control mosquito larvae.

## 1. Introduction

The escalating prevalence of insecticide resistance presents a substantial and growing obstacle to effective mosquito control programs, often rendering established active ingredients and commercial formulations less effective. This problem of insecticide resistance is of considerable public health significance when there are no vaccines or prophylactic treatments available for a mosquito-borne disease; thereby, communities are reliant on mosquito abatement to curb transmission and disease outbreaks. Successful mosquito control relies on a multi-pronged approach, termed integrated mosquito management (IMM), which may include surveillance, community engagement, biological control, larval source reduction, and the targeted application and evaluation of larvicides and adulticides [1]. Larvicides are often a favored chemical control option due to the diverse range of active ingredients with distinct modes of action and varied formulations. Commonly used larvicides, often called biorational for their minimal off-target effects, are generally classified into two groups: microbial agents and insect growth regulators (IGRs).

Some IGRs mimic juvenile hormones, termed juvenile hormone analogs (JHAs), which interrupt successful metamorphosis and result in death when exposed in the larval stage [2,3]. JHAs are highly target-specific [4] and have prolonged residual activity, binding to organic and inorganic materials in larval habitats [5]. As a result, JHAs are widely used in mosquito control programs around the world, particularly in human-made larval habitat sources like stormwater catch basins. In the United States, two JHAs are labeled for use in stormwater catch basins: S-methoprene and pyriproxyfen. S-methoprene was originally labeled for larvicide use in 1975 and has been in widespread use since [6]. In the Chicago, IL region, S-methoprene-based products have been used in stormwater catch basins for 10–20+ years, in the absence of product rotation, to control *Culex pipiens* and *Cx. restuans*, the local West Nile virus (WNV) vectors [7]. This history of widespread, continuous, and uninterrupted selective pressure has resulted in significantly reduced susceptibility to S-methoprene in local *Cx. pipiens* populations [7,8].

Insecticide cross-resistance occurs when a population that has developed resistance to one insecticide subsequently exhibits reduced susceptibility and resistance to other insecticides, even without direct exposure to the latter compounds. Cross-resistance occurs because the underlying resistance mechanism confers protection against multiple insecticides, even insecticides with dissimilar modes of action. Fully characterizing cross-resistance with S-methoprene resistance is essential to IMM programs in the Chicago metropolitan area and other regions where S-methoprene-tolerant mosquitoes are present. In a laboratory-selected strain, S-methoprene-resistant *Cx. quinquefasciatus* were slightly cross-resistant to spinosad, fipronil, indoxacarb, and pyriproxyfen and highly cross-resistant to *Lysinibacillus sphaericus* (*Ls*) [9]. Observations in laboratory strains are not always indicative of what occurs in naturally occurring mosquito populations due to genetic bottlenecks and inbreeding [10]. For example, our investigation in 31 field-collected S-methoprene-resistant *Cx. pipiens* populations revealed this cross-resistance relationship with *Ls* may be broadly erroneous outside the laboratory [8]. We reasoned that further investigations are necessary to understand if the cross-resistance observed in this laboratory is present for each of the aforementioned active ingredients. Pyriproxyfen in particular is of interest because it is an IGR with an identical mode of action to S-methoprene and was more recently labeled for mosquito control use, leading to subsequent uptake from mosquito control districts in the Chicago metropolitan area to control S-methoprene-resistant *Culex*. In the absence of cross-resistance, pyriproxyfen has a long half-life and high binding affinity [5,11], potentially resulting in sublethal concentrations in stormwater catch basins after product applications, leading to decreased susceptibility in mosquitoes and warranting investigation.

To understand the potential for a cross-resistant relationship between S-methoprene and pyriproxyfen in local *Cx. pipiens* populations, in this study, we aimed to (1) assess resistance to S-methoprene, (2) quantify susceptibility to pyriproxyfen, and (3) determine if resistance to S-methoprene correlated with pyriproxyfen resistance. We evaluated 31 *Cx. pipiens* populations from two mosquito abatement districts in the Chicago, IL metropolitan area, where S-methoprene tolerance is documented. All populations assessed were collected from areas with a long-term history of S-methoprene use but with varying histories of pyriproxyfen use. We hypothesized that S-methoprene-resistant populations would be cross-resistant to pyriproxyfen, even without prior exposure or selection pressure from pyriproxyfen use. Ultimately, we aimed to determine whether the presence of S-methoprene cross-resistance precludes the use of pyriproxyfen in operational mosquito control programs and how S-methoprene resistance influences resistance management strategies.

## 2. Materials and Methods

### 2.1. Collection Sites

**Figure 1:**
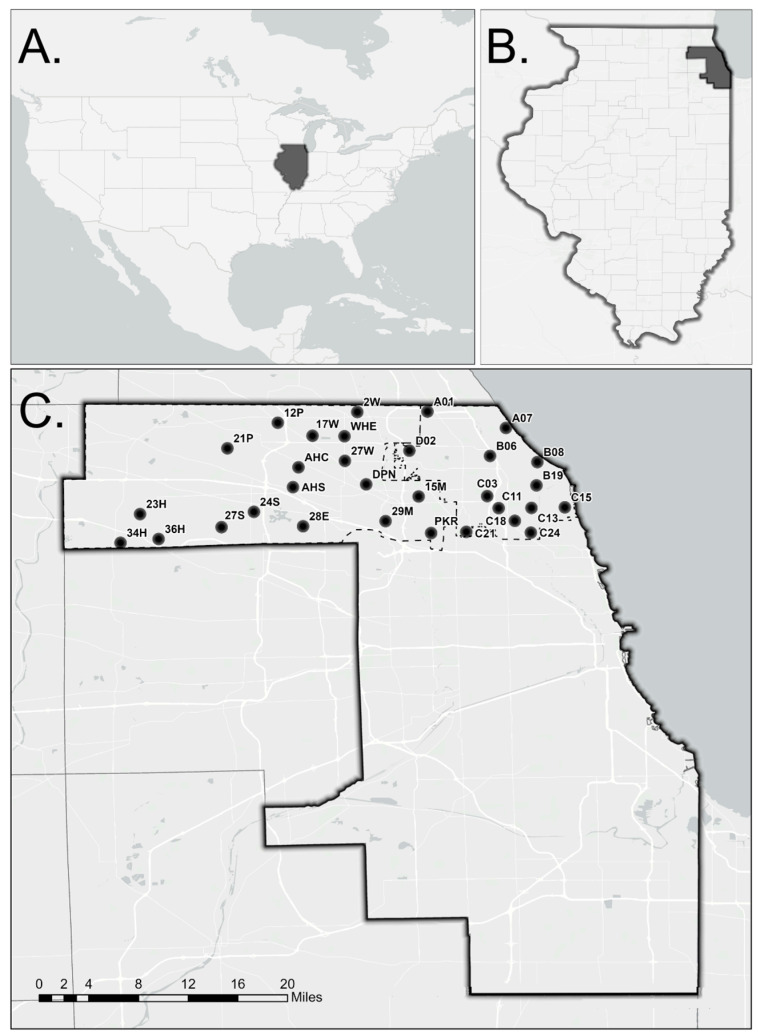
Map of study area. (**A**) United States of America, highlighting Illinois; (**B**) Illinois, highlighting Cook County; (**C**) Collection sites within Cook County. Map created by Austin Robak using Esri basemap data © OpenStreetMap contributors, Esri, under CC BY 4.0 (https://creativecommons.org/licenses/by/4.0/, accessed on 31 October 2025).

*Culex pipiens* egg rafts were collected from 31 sites across two mosquito control districts in the north and northwestern suburbs of Chicago, IL, USA (Figure 1, Supplementary Table S1). Each collection site encompassed approximately 2.3 km^2^ and was separated from other sites by at least 1.6 km. Collection sites in the North Shore Mosquito Abatement District (NSMAD) include Northfield (B06), Northbrook (A01), Glencoe (A07), Glenview (D02), Wilmette (B08), Skokie (C11, C13, C18), Evanston (B19, C15), Morton Grove (C03), Niles (C21), and Lincolnwood (C24). Sites from the Northwest Mosquito Abatement District (NWMAD) include Des Plaines (DPN, 15M, 29M), Elk Grove (28E), Park Ridge (PKR), Bartlett (34H), Hanover Park (36H), Streamwood (23H), Schaumburg (24S, 27S), Arlington Heights (AHC, AHS, 17W), Prospect Heights (27W), Wheeling (WHE, 2W), and Palatine (12P, 21P).

### 2.2. Mosquito Collection and Rearing

Egg rafts were collected from three to five gravid ovitraps per site, baited with an alfalfa pellet infusion. Collections were conducted July through September 2025 (epidemiological weeks 27–39). Each site was sampled one to three times on sequential days until a sufficient quantity of egg rafts was collected. A minimum of 12 egg rafts was required per collection site. Egg rafts were hatched in 6 oz Styrofoam cups (Item 6SJ12, Dart Container Company, Mason, MI, USA) with ~150 mL of tap water. Once mosquitoes developed to the second instar, *Cx. pipiens* larvae were identified morphologically [12] and pooled by collection site. Other *Culex* spp. larvae were discarded. Larvae were fed ground TetraMin^®^ tropical fish flakes (Spectrum Pet Brands LLC, Blacksburg, VA, USA) and reared under standard conditions (27 °C, 80% RH, 16:8 light:dark cycle) until they reached late fourth instar.

### 2.3. Larvicide Bioassays

Laboratory larvicide bioassays were conducted according to previous methods [7,13,14]. Briefly, approximately 20–25 late fourth instar larvae were transferred to a 6 oz styrofoam cup with 100 mL tap water and 100 mg rabbit pellets (Kaytee Specialty Products Inc., Clinton, WI, USA). Technical-grade S-methoprene and pyriproxyfen (Items 33375 and 34175, Sigma-Aldrich, St. Louis, MO, USA) were diluted separately in series with analytical-grade acetone (Item 270725, Sigma-Aldrich, St. Louis, MO, USA) to generate stock solutions. Solutions were stored in borosilicate amber glass at 4 °C and acclimated to room temperature before use. Each field-collected population and susceptible control mosquitoes were subjected to a minimum of three replicates for each concentration and active ingredient tested. When a surplus of larvae was available, additional replicates and/or concentrations were tested. Approximately half of the reared larvae were used in S-methoprene assays, while the other half were used in pyriproxyfen assays, so larvae from the same egg rafts, of presumed similar genetics, were exposed to both active ingredients. In total, each population was exposed to 3–8 replicates of 9–15 concentrations of S-methoprene (Supplementary Tables S2 and S3) and 3–8 replicates of 10–18 concentrations of pyriproxyfen (Supplementary Tables S4 and S5). Final exposure concentrations ranged from 5 × 10^−4^ ppb to 5 × 10^3^ ppb for S-methoprene and 1 × 10^−6^ ppb–1 × 10^3^ ppb for pyriproxyfen. Untreated control cups were implemented in triplicate and in parallel with larvicide-treated cups for all field-collected populations and the susceptible strain. Bioassay cups were covered with a modified lid [7] and maintained under ambient conditions (23 °C, 60% RH) on lab benches to avoid contamination of rearing facilities. Results were recorded after all mosquitoes had either emerged successfully or died. Dead larvae, dead pupae, and incompletely emerged adults were combined in mortality counts, also termed emergence inhibition.

### 2.4. Analysis

Mortality was corrected with Abbott’s formula [15]. A probit analysis [16,17] was used to calculate LC_50_ and LC_90_ values, with 95% confidence intervals, for each population. A Pearson’s χ^2^ goodness-of-fit test and H (heterogeneity factor) were used to evaluate model fit. Resistance ratios (RRs) were calculated by dividing the LC_50_ or LC_90_ of each field-collected population by the respective value for the susceptible strain (COL), resulting in RR_50_ and RR_90_ values. Field-collected populations were categorized for resistance intensity as follows: susceptible (RR < 5), moderate resistance (5 < RR < 10), high resistance (10 < RR < 100), and extreme resistance (RR > 100) [7,18]. There are no previously established resistance categories from the WHO for *Culex* genus mosquitoes; the thresholds for *Aedes* genus mosquitoes were used [18].

The relationship between S-methoprene and pyriproxyfen resistance was explored with gamma regression with a log link, as the data were not normally distributed, non-negative non-integers, and were over-dispersed. We hypothesized that the level of pyriproxyfen resistance would correlate with the level of S-methoprene resistance and the amount of pyriproxyfen pressure from mosquito control activities. Separate models were created for RR_50_ and RR_90_ data sets. The response variable with the pyriproxyfen RR_50_ or RR_90_ values. The explanatory variables considered included the S-methoprene RR_50_ or RR_90_ values and the number of seasons where all catch basins were treated with pyriproxyfen from each collection site. Model selection was completed by comparing Akaike information criterion (AIC) and Nagelkerke R^2^ values [19]. All analyses were completed and visualized using R studio, version 4.5.1 (R Development Core Team 2025) with packages ‘ecotox’, ‘MuMIn’, ‘MASS’, and ‘ggplot2’ [20,21,22,23].

## 3. Results

**Figure 2:**
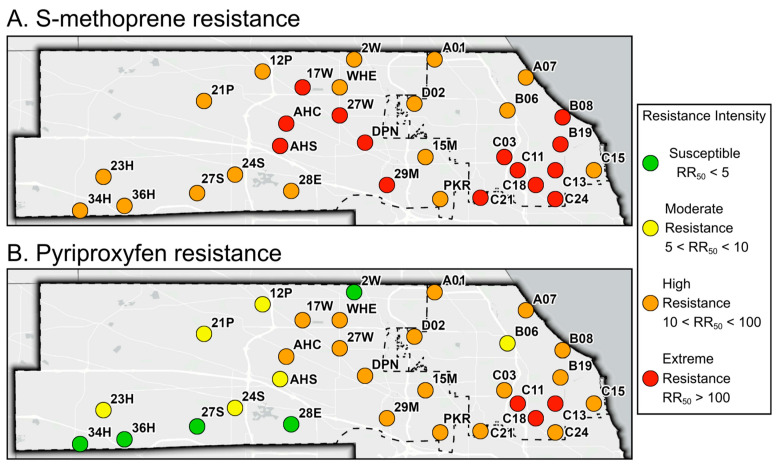
Resistance status of each field-collected *Cx. pipiens* population to S-methoprene (**A**) and pyriproxyfen (**B**) at RR_50_. Resistance intensity categories according to the WHO [18]. Map created by Austin Robak using Esri basemap data © OpenStreetMap contributors, Esri, under CC BY 4.0 (https://creativecommons.org/licenses/by/4.0/, accessed on 31 October 2025).

All 31 field-collected *Cx. pipiens* populations were at least highly resistant (RR_50_ > 10) to S-methoprene (Figure 2, Supplementary Table S6) [18]. Of these 31 populations, 14 displayed an ‘extreme’ resistance to S-methoprene (RR_50_ > 100) [7]. When examining the S-methoprene RR_90_ values, two were susceptible or had low levels of resistance, six were moderately resistant, 17 were highly resistant, and six were extremely resistant (Supplementary Table S6) [7,18]. In total, 26 of the 31 populations studied were at least moderately resistant (RR_50_ > 5) to pyriproxyfen; specifically, at RR_50_, five populations were susceptible or had low resistance, six populations were moderately resistant, 17 were highly resistant, and three were extremely resistant (Figure 2 and Supplementary Table S7). Increased susceptibility to pyriproxyfen was observed at RR_90_, where 16 populations were susceptible or had low resistance, seven were moderately resistant, seven were highly resistant, and one was extremely resistant (Supplementary Table S7). The susceptible control strain of *Cx. pipiens* was susceptible to both S-methoprene and pyriproxyfen in alignment with previous work with this same colony [7,8], as well as other published literature [10,13,14].

**Figure 3:**
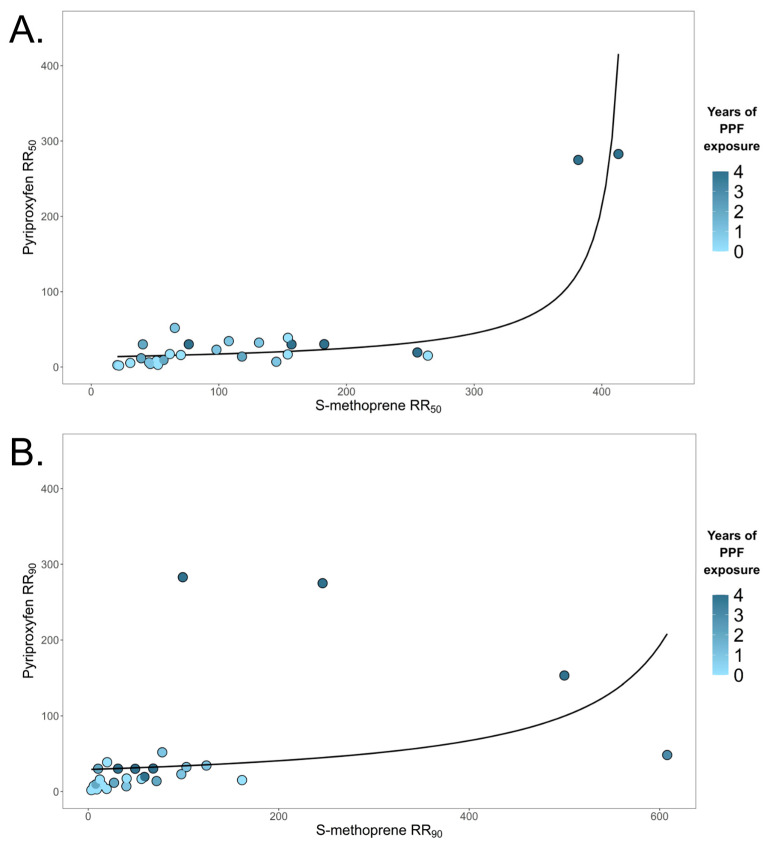
Comparison of resistance to S-methoprene and pyriproxyfen in 31 field-collected populations of *Cx. pipiens*. (**A**) Resistance ratios calculated for LC_50_ values and (**B**) using LC_90_ values. Color indicates the number of pyriproxyfen treatments in the collection site. The trend line follows the log-linked gamma regression pyriproxyfen RR~S-methoprene RR.

Gamma regression analysis revealed that the level of pyriproxyfen resistance was significantly correlated to both the level of S-methoprene resistance and the number of pyriproxyfen applications made to catch basins at both RR50 and RR_90_ (Table 1 and Figure 3). Final model selection included both the level of S-methoprene resistance and the number of pyriproxyfen catch basin applications for both models (Supplementary Table S8). The estimates for S-methoprene RR50 were 1.01, indicating that pyriproxyfen resistance increases by 1% per unit increase in S-methoprene resistance. For example, if S-methoprene RR_50_ is 200, then pyriproxyfen RR_50_ would be 202. In addition, the number of pyriproxyfen catch basin applications significantly increases pyriproxyfen resistance. Each additional treatment of pyriproxyfen increased the resistance ratio by 25% and 53% at RR_50_ and RR_90_, respectively, though this was not significant at RR_50_. Indeed, all populations that had three to four applications were all highly resistant to pyriproxyfen (RR_50_ > 10). It is important to remember that the increases in resistance are not linear, as this relationship follows a gamma distribution and estimates are multiplicative.

## 4. Discussion

In this study, we demonstrated that S-methoprene resistance correlates with pyriproxyfen resistance, indicating a potential cross-resistance relationship. We detected high levels of S-methoprene resistance (RR_50_ > 10) in all 31 populations studied, of which 26 exhibited pyriproxyfen resistance (RR_50_ > 5) (Figure 2). Increased levels of S-methoprene resistance ratios resulted in equally increased levels of pyriproxyfen resistance ratios; the incidence rates were equal (Table 1). The number of pyriproxyfen applications was associated with increased levels of pyriproxyfen resistance, but it was not necessary for reduced susceptibility to be present. Indeed, 8 of 11 populations exhibited resistance to pyriproxyfen even though zero applications were made in the area (Figure 2, Supplementary Table S7). However, it is critical to understand that if a population is resistant to S-methoprene, this does not guarantee resistance to pyriproxyfen. For example, five populations that were highly resistant to S-methoprene were not resistant to pyriproxyfen at either RR_50_ or RR_90_ (Figure 2, Supplementary Tables S6 and S7). Therefore, if a population is resistant to S-methoprene, it is possible that pyriproxyfen resistance is also present, but it is not guaranteed.

To the best of our knowledge, this is the first report of widespread high levels of resistance to pyriproxyfen in a medically significant mosquito species, though this is not due to a lack of surveillance. Susceptibility to high levels of resistance to pyriproxyfen has been documented in field-collected populations of *Aedes* species [24,25,26,27,28,29,30,31,32,33,34,35,36], though minimal resistance has been detected in *Culex* species [24,37,38,39,40]. The highest level of pyriproxyfen resistance reported was in one *Ae. aegypti* population from California with RR_50_ value of 38.7 [36]. In the current study, we report 20 populations with at least a high level of resistance (RR_50_ > 10), with three populations with RR_50_ values greater than 100 (Figure 2, Supplementary Table S7). Conversely, reduced susceptibility to S-methoprene in *Culex* species has been reported around the world [13,14,41,42,43,44], including our own investigations [7,8]. With this repeated sampling, many populations from the Chicago region are consistently among the highest reported, exhibiting resistance year after year [7,8].

Cross-resistance between these two active ingredients is not unexpected, as both active ingredients are juvenile hormone analogs (JHAs). Therefore, it can be assumed that the resistance mechanisms evolved to combat one JHA work on others. Although the molecular mechanisms underlying JHA resistance remain poorly characterized, non-specific mechanisms like increased metabolic detoxification and decreased absorption are hypothesized [45,46,47]. Enhanced metabolic detoxification is a compelling resistance mechanism, as many of these *Cx. pipiens* populations are resistant to a variety of other insecticides, including pyrethroids, organophosphates, and *Ls* [7,8,48,49,50,51]. This predisposition for broad metabolic activity may be further amplified by the highly organic nature of the urban larval habitats these mosquitoes occupy, pre-selecting for elevated detoxification capacity [52].

It is possible that pyriproxyfen resistance pressures originated from sources outside the activity of mosquito abatement districts. Pyriproxyfen is formulated to control a variety of insect pests in a multitude of habitats. For example, pyriproxyfen can be found in a multitude of general- and restricted-use products to control lepidopteran, coleopteran, blattodean, orthopteran, siphonapteran, and dipteran pests in and around human-made environments. It is possible that these products accidentally end up in catch basins from improper use, runoff, or improper disposal, leading to sublethal exposures. An assortment of household and industrial chemicals, including insecticides, can be found in catch basins, adding additional selection pressures [53].

In this investigation, there were no field-collected *Cx. pipiens* populations from the Chicago, IL, USA metropolitan area that were susceptible to both S-methoprene and pyriproxyfen, nor were there any populations that were tolerant to pyriproxyfen but susceptible to S-methoprene. It is unclear if reduced susceptibility to only pyriproxyfen would reciprocally cause cross-resistance to S-methoprene, but it is probable based on the resistance mechanisms. Further investigations are needed to understand this potential reciprocal relationship.

The association between resistance ratios observed in laboratory assays and the correlation to real-world commercial product effectiveness remains ambiguous and warrants further investigation. An elevated RR suggests that a population possesses genetically reduced susceptibility to a given active ingredient, which implies a high potential for reduced field efficacy or even control failure, but this assumption may not translate in operational settings. For example, both the NSMAD and the NWMAD continue to use pyriproxyfen in mosquito control operations and have not observed a decrease in effectiveness when applied at full label rates. It is possible that applying pyriproxyfen-based larvicides at lower label rates could result in product failures, but more research is needed to make this determination. Formulated products can be applied at higher rates, depending on the label, to overcome resistance and often contain inert ingredients that increase potency and residual time. Indeed, the LC_50_ and LC_90_ doses of pyriproxyfen are much lower than the doses of S-methoprene (Supplementary Tables S6 and S7); thus, resistance can be overcome in the field. In general, if resistance is detected in field-collected mosquito populations, increasing application rates is recommended to reduce sublethal exposures. Further work investigating product effectiveness on resistant populations is necessary to understand the genuine risk of resistance to mosquito control operations and the protection of public health.

Currently, there are no proven methodologies for reducing S-methoprene or pyriproxyfen resistance in mosquitoes. Common resistance management tactics include product rotation with different active ingredients with different modes of action, combining active ingredients, or applying products in a mosaic pattern, allowing for untreated refugia [18]. It is unclear if removing the selection pressure will guarantee increased susceptibility. Despite these unknowns, mosquito control professionals are still tasked with reducing mosquito populations and mitigating the threat of vector-borne disease. Rotation between or combining S-methoprene and pyriproxyfen is an ill-advised tactic to reduce JHA resistance due to cross-resistance and an identical mode of action. Other resistance management techniques, like rotating and combining a JHA with larvicide products with distinct modes of action, like spinosad, *Ls*, or *Bacillus thuringiensis israelensis* (Bti)*,* should be effective despite low levels of cross-resistance, but this has yet to be validated.

Overall, we demonstrated that pyriproxyfen cross-resistance may occur in S-methoprene-resistant *Cx. pipiens* populations, regardless of prior pyriproxyfen exposure, though it is not guaranteed. Understanding larvicide resistance, cross-resistance, and interactions between active ingredients is critical for mosquito control operations to make informed product selection choices and to practice insecticide resistance management. These results underscore the need for routine larvicide resistance surveillance where larvicides are used for mosquito control and for further research in effective resistance management tactics.:

## Figures and Tables

**Table 1:** Final gamma regression models for pyriproxyfen resistance. Estimates (exponentiated), 95% confidence intervals (CI), and *p*-values are reported.

	Pyriproxyfen RR_50_			Pyriproxyfen RR_90_		
Predictors	Estimates	CI	*p*	Estimates	CI	*p*
(Intercept)	7.94	5.12–12.73	<0.001	10.00	6.22–17.03	<0.001
S-methoprene RR_50_	1.01	1.00–1.01	<0.001			
Number of pyriproxyfen applications	1.25	0.97–1.63	0.065	1.53	1.21–2.00	0.001
S-methoprene RR_90_				1.00	1.00–1.01	0.032
Observations	31			31		
R^2^ Nagelkerke	0.817			0.746		

## Data Availability

The original contributions presented in this study are included in the article/Supplementary Materials. Further inquiries can be directed to the corresponding author.

## Supplementary Materials

The following supporting information can be downloaded at https://www.mdpi.com/article/10.3390/insects17030241/s1. Table S1: GPS coordinates of ovitraps per collection site. Table S2: Number of replicates per collection site and per concentration of S-methoprene. Susceptible colony mosquitoes are denoted by COL. Untreated controls for mortality correction are listed as ‘control’. Table S3: Raw bioassay mortality data for S-methoprene. Table S4: Number of replicates per collection site and per concentration of pyriproxyfen. Susceptible colony mosquitoes are denoted by COL. Untreated controls for mortality correction are listed as ‘control’. Table S5: Raw bioassay mortality data for pyriproxyfen. Table S6: Summary of S-methoprene dose response data, resistance ratios, and statistical outputs for field-collected *Cx. pipiens*. Table S7: Summary of pyriproxyfen dose response data, resistance ratios, and statistical outputs for field-collected *Cx. pipiens*. Table S8: Model selection table.

## Abbreviations

The following abbreviations are used in this manuscript:

WNV	West Nile virus
IMM	Integrated mosquito management
IGR	Insect growth regulator
JHA	Juvenile hormone analog
RR	Resistance ratio
Ls	*Lysinibacillus sphaericus*
Bti	*Bacillus thuringiensis israelensis*
NSMAD	North Shore Mosquito Abatement District
NWMAD	Northwest Mosquito Abatement District
