# Supplementary material for: Resistance to S-Methoprene Correlates with Pyriproxyfen Resistance in Field-Collected Culex pipiens
Source: Insects. 2026 Feb 26;17(3):241. doi: 10.3390/insects17030241 (PMC13027244; doi:10.3390/insects17030241)
Supplement: Supplementary file 1 [file insects-17-00241-s001.zip › Supplementary Table S1.pdf]

Supplementary Table S1. GPS coordinates of ovitraps per collection site.

Collection Site	Ovitrap Locations				
23H	42.0271, -88.1809	42.0183, -88.1838	42.0206, -88.1754	42.0194, -88.1695	42.0284, -88.1704
34H	41.9943, -88.2033	41.9899, -88.1998	41.9938, -88.1937	41.9887, -88.1927	
36H	41.9998, -88.1543	41.9976, -88.1535	41.9931, -88.157	41.9979, -88.1462	
24S	42.0221, -88.0487	42.0282, -88.0432	42.0246, -88.037	42.0282, -88.0431	42.0306, -88.0389
27S	42.0187, -88.0737	42.0135, -88.0763	42.0116, -88.0767	42.0074, -88.0775	42.0058, -88.0847
28E	42.0145, -87.9906	42.0065, -87.9878	42.0077, -87.9796	42.0115, -87.9796	42.0156, -87.9796
DPN	42.0636, -87.9132	42.0633, -87.9065	42.0554, -87.9128	42.0408, -87.8652	
15M	42.0493, -87.8632	42.0495, -87.8579	42.0428, -87.849	41.9975, -87.8321	
PKR	42.0075, -87.8411	42.0067, -87.8262	41.9972, -87.8353	42.0133, -87.8943	
29M	42.0216, -87.8901	42.0169, -87.8845	42.0148, -87.8905	42.1406, -87.9283	
2W	42.1489, -87.9109	42.1468, -87.9268	42.1423, -87.9137	42.1169, -87.9456	
WHE	42.1161, -87.9317	42.1100,-87.9327	42.1111, -87.946	42.0856, -87.9383	
27W	42.0922, -87.9342	42.0918, -87.9377	42.0881, -87.9298	42.0854, -88.0022	42.0817, -87.9414
AHC	42.0768, -87.9930	42.0819, -87.9988	42.0802, -88.0025	42.1222, -87.9733	
AHS	42.0542, -87.9930	42.0597, -87.9987	42.0603, -88.0007	42.1254, -88.0179	
17W	42.1100, -87.9756	42.1135, -87.967	42.1203, -87.9706	42.0974, -88.073	
12P	42.1352, -88.0095	42.1354, -88.0143	42.129, -88.0145	42.0953, -87.8609	
21P	42.1093, -88.0754	42.1092, -88.0676	42.103, -88.0692	42.1432, -87.83	

D02	42.1043, -87.8582	42.1030, -87.8619	42.1019, -87.8651	42.0856, -87.766	42.0965, -87.8564
A01	42.1500, -87.8475	42.1512, -87.8451	42.147, -87.8301	42.0817, -87.7126	
A07	42.1249, -87.7516	42.1217, -87.7477	42.1183, -87.7432	42.0554, -87.7045	
B06	42.0994, -87.7721	42.0954, -87.7644	42.093, -87.7608	42.0469, -87.7786	42.0949, -87.7702
B08	42.0956, -87.7253	42.0904, -87.7211	42.0857, -87.7159	42.0319, -87.7181	
B19	42.0637, -87.7241	42.0586, -87.7227	42.0606, -87.7112	42.0321, -87.6704	42.0543, -87.7065
C03	42.0511, -87.7627	42.0476, -87.7628	42.0494, -87.776	42.0158, -87.744	
C11	42.0401, -87.7590	42.0328, -87.7520	42.0282, -87.7597	42.0103, -87.7973	
C13	42.0373, -87.7105	42.0355, -87.7221	42.0330, -87.724	42.001, -87.7251	42.0290, -87.7256
C15	42.0311, -87.6858	42.0324, -87.6813	42.032, -87.6756	42.032, -87.6756	
C18	42.0207, -87.7457	42.0205, -87.7381	42.013, -87.7389	42.013, -87.7389	
C21	42.0026, -87.7902	42.0060, 87.7931	42.0082, -87.7962	42.0082, -87.7962	
C24	42.0097, -87.7184	42.0043, -87.7180	41.9985, -87.7187	41.9985, -87.7187	41.9983, -87.7268
