# Supplementary material for: Resistance to S-Methoprene Correlates with Pyriproxyfen Resistance in Field-Collected Culex pipiens
Source: Insects. 2026 Feb 26;17(3):241. doi: 10.3390/insects17030241 (PMC13027244; doi:10.3390/insects17030241)
Supplement: Supplementary file 1 [file insects-17-00241-s001.zip › Supplementary Table S2.pdf]

Supplementary Table S2. Number of replicates per collection site and per concentration of S-methoprene. Susceptible colony mosquitoes denoted by COL. Untreated controls for mortality correction are listed as "control."

Collection Site	**Concentration of S-methoprene (ppb)**																				
	0.0005	0.001	0.005	0.01	0.05	0.075	0.1	0.5	0.75	1	5	7.5	10	50	75	100	500	750	1000	5000	Control	**Total**
12P							3	4	4	3	4	4	3	4	4	3	4	4	3		9	**56**
15M				4			3	3	4	4	3	4	4	3	4	4	3	3	3	3	12	**64**
17W				3			3	3	4	3	3	4	3	3	4	3	3	4	3	3	9	**58**
21P							4	4	3	3	4	3	3	4	3	3	4	3	3		12	**56**
23H				3	3	3	3	3	3	3	3	3	3		3	3			3		6	**45**
24S								3	3	3	3	3	3	3	3	3	3	3	2		9	**44**
27S							3	4	3	3	3	3	3	3	3	4	3	3	3		9	**49**
27W							3	3	3	3	3	3	3	3	3	3	3	3	3		9	**48**
28E							3	3	3	3	3	3	3	3		3	3		3		7	**40**
29M				3			3	3	3	4	3	4	4	3	4	3	3	3	4	3	9	**59**
2W							3	4	3	3	4	3	3	4	3	3	3	3	3		9	**51**
34H				3	3	3	3	3	3	3	3	3	3	3	3	3	3	3	3		9	**57**
36H				2	3	3	3	3	3	3	3	3	3	3	3	3	3	3	3		9	**56**
A01							3	3	3	5	3	3	4	3	3	3	3	3	3		9	**51**
A07							3	5	3	4	6	3	4	3	3	4	3	3	3	3	6	**56**
AHC				3	3	3	3	3	3	4	3	4	4	3	3	4	3	3	3	3	9	**64**
AHS				2	3		3	4	3	4	4	3	4	4	3	3	3	3	3	3	9	**61**
B06					3		3	3	3	3	3	3	3	3	3	3	3	3	3		9	**51**
B08							4	3	3	5	3	3	5	3	3	4	3	3	4		9	**55**
B19							4	4	2	4	4	4	4	4	3	5	4	3	4		6	**56**
C03							4	4	3	3	4	3	3	4	3	4	4		4	4	10	**57**
C11							3	4	6	3	4	6	3	4	6	3	4	6	3		11	**66**
C13							4		4	4		4	4		4	4		3	4		7	**42**
C15				3			3	4	3	4	4	3	4	4	3	2	4		4	3	9	**57**
C18				4			4	5	3	4	5	3	4	5	3	4	5	3	4	3	6	**65**
C21				2			3	4	3	4	4	3	4	4	3	4	4	3	3	3	9	**60**
C24				3			3	3	3	3	4	3	4	6	3	6	4		4	3	9	**61**
Colony	4	4	3	4	8	7	4	9	8	5	6	8	5	6	7		3				9	**100**
D02				3		3	3	3	4	3	3	4	3	3	3	3	3	3	3		9	**56**
DPN							3	3	3	3	4	3	4	4	3	4	3	3	4	3	6	**53**
PKR							3	3	3	4	4	3	4	4	3	4	3	3	4	3	6	**54**
WHE							3	3	3	3	4	3	4	4	3	4	3	3	4	3	6	**53**
**Total**	**4**	**4**	**3**	**42**	**26**	**23**	**100**	**113**	**108**	**113**	**114**	**112**	**115**	**110**	**105**	**109**	**100**	**83**	**103**	**43**	**272**	**1801**
