# Supplementary material for: Resistance to S-Methoprene Correlates with Pyriproxyfen Resistance in Field-Collected Culex pipiens
Source: Insects. 2026 Feb 26;17(3):241. doi: 10.3390/insects17030241 (PMC13027244; doi:10.3390/insects17030241)
Supplement: Supplementary file 1 [file insects-17-00241-s001.zip › Supplementary Table S3.pdf]

Supplementary Table S3. Number of replicates per collection site and per concentration of pyriproxyfen. Susceptible colony mosquitoes denoted by COL. Untreated controls for mortality correction are listed as “control.”

Collection Site	Concentration of Pyriproxyfen (ppb)																						
	1E-06	1E-05	0.0001	0.0005	0.0008	0.001	0.005	0.0075	0.01	0.05	0.075	0.1	0.5	0.75	1	5	7.5	10	50	100	500	1000	Control	**Total**
12P			3	4		3	4	3	3	4	3	3	4	3	3	4	3	3		4			9	**63**
15M			3	3	3	3	3	3	4	3	3	4	3	3	4	3	3	3	3	3			6	**63**
17W			3	3		3	3	4	3	3	4	3	3	4	3	3	4	3		3			9	**61**
21P			3			3	3	3	3	3	3	3	3	3	3	3	3	3		3			9	**54**
23H			3		3	3	3	3	3	3	3	3	3	3	3			3					6	**45**
24S			3		3	3	3	3	3	3	3	3	3	3	3	3		3		3			9	**51**
27S			3	4		3	3	3	3	3	3	3	3	3	3	3	3	3		3			9	**56**
27W			3			3	3	3	3	3	3	3	3	3	3	3	3	3					9	**51**
28E			3			3	3	3	3	3	3	3	3		3	3		3					7	**43**
29M			3	3		3	3	3	4	3	3	4	3	3	4	3	3	3	3	3			9	**63**
2W			3			3	4	3	3	4	3	3	4	3	3	3	3	3		3			9	**57**
34H			3	3	3	3	3	3	4	3	3	3	3	3	3	3	3	3					9	**58**
36H			3	3	3	3	3	3	3	3	3	3	3	3	3	3	3	3					9	**57**
A01			4	3	3	4	3	3	4	3	3	3	3	3	2	3		1					9	**54**
A07			3	3		4	3	3	4	3	3	4		3	4		3	3		3			6	**52**
AHC			3	3	3	3	3	3	3	3	3	3	3	3	3	3	3	3	3	3			9	**63**
AHS			3	3		3	4	3	4	4	3	3	4	3	3	3	3	3	3	3			9	**64**
B06			3	3		3	3	3	3	3	3	3	3	3	3	3	3	3					9	**54**
B08			4	3		4	3	3	4	3	3	4	3	3	3	3		3		4			6	**56**
B19			4	3	3	4	4	3	4	5	3	4	4	3	4	5		4		4			6	**67**
C03			4	3		4	4	3	4	3	3	4	4	3	4	3		4		4			9	**63**
C11			3		3	3	3	6	3	3	6	3	3	6	3	3	6	3					7	**64**
C13			3			4		4	4		4	4		4	4		4	6					5	**46**
C15			4			4	4		4	4	3	4	4	4	4	3	3	4	4	4			6	**63**
C18			4	4		4	4	3	4	4	5	4	4	4	4	4	4	4	4	4	3	3	9	**83**
C21			3	3		3	4	3	4	4	3	4	4	3	5	4	3	3	3	4			9	**69**
C24			4			4	3		4	4	3	4	5	3	5	5	3	4	4	4			9	**67**
Colony	3	8	8		4	8	3	4	8	2	4	8	4	4	3								9	**79**
D02		3	3	3	3	3	3	3	3	3	3	3	3	3	3	3	3	3					9	**60**
DPN			3	3		3	3		3	3	3	4	3	3	4	3	3	3	3	3			6	**56**
PKR			3	3		3	3		3	3	3	4	3	3	4	3	3	4	3	4			6	**58**
WHE			3	3		3	3		3	3	3	4	3	3	4	4	3	4	3	3			6	**57**
**Total**	**3**	**11**	**108**	**66**	**34**	**110**	**101**	**87**	**115**	**101**	**104**	**115**	**101**	**101**	**110**	**92**	**78**	**101**	**36**	**72**	**3**	**3**	**253**	**1897**
