# Supplementary material for: Resistance to S-Methoprene Correlates with Pyriproxyfen Resistance in Field-Collected Culex pipiens
Source: Insects. 2026 Feb 26;17(3):241. doi: 10.3390/insects17030241 (PMC13027244; doi:10.3390/insects17030241)
Supplement: Supplementary file 1 [file insects-17-00241-s001.zip › Supplementary Table S6.pdf]

Supplementary Table S6. Summary of S-methoprene dose response data, resistance ratios, and statistical outputs for field-collected *Cx. pipiens*.

Collection Site	N(n)[a]	N PPF[b]	Slope ± s.e.m.	P-value[c]	H (χ^2/df)	LC_{50}[d] (95% CI)	LC_{90}[d] (95% CI)	RR_{50} (95% CI)	RR_{90} (95% CI)
COL	91 (2003)	0	0.67 ± 0.03	1.87E-35	4.12	0.11 (0.06 - 0.18)	9.01 (4.69 - 21.17)	-	-
12P	47 (985)	0	1.09 ± 0.06	3.81E-66	9.77	3.41 (1.49 - 7.05)	51.51 (21.32 - 242.36)	30.61 (13.37 - 63.21)	5.71 (2.36 - 26.87)
15M	52 (1074)	1	0.67 ± 0.04	5.60E-09	2.59	10.94 (6.58 - 18.07)	882.10 (397.87 - 2592.20)	98.14 (59.03 - 162.04)	97.79 (44.11 - 287.38)
17W	49 (1019)	0	0.76 ± 0.04	1.04E-21	4.37	29.41 (15.83 - 55.64)	1456.70 (567.72 - 5880.16)	263.74 (141.99 - 498.98)	161.49 (62.94 - 651.91)
21P	44 (949)	0	1.34 ± 0.07	3.11E-09	2.82	5.69 (3.99 - 8.07)	51.29 (32.14 - 95.84)	51.03 (35.83 - 72.36)	5.69 (3.56 - 10.63)
23H	39 (788)	0	0.94 ± 0.06	5.59E-09	2.93	5.70 (3.46 - 9.84)	132.21 (60.09 - 413.00)	51.15 (31.09 - 88.28)	14.66 (6.67 - 45.79)
24S	35 (752)	0	1.15 ± 0.07	3.54E-03	1.79	5.05 (3.56 - 7.04)	65.46 (41.44 - 118.52)	45.28 (31.95 - 63.19)	7.26 (4.59 - 13.14)
27S	40 (867)	1	1.03 ± 0.06	1.78E-10	3.16	2.29 (1.38 - 3.63)	40.17 (21.93 - 93.36)	20.53 (12.35 - 32.62)	4.45 (2.43 - 10.35)
27W	39 (825)	0	0.91 ± 0.05	2.82E-04	1.78	26.42 (17.82 - 39.40)	683.18 (374.49 - 1506.74)	154.08 (86.32 - 273.91)	19.84 (9.78 - 58.41)
28E	33 (678)	1	1.07 ± 0.07	3.00E-07	2.81	5.17 (3.26 - 8.23)	80.70 (41.65 - 211.53)	46.34 (29.24 - 73.84)	8.95 (4.61-23.45)
29M	50 (1051)	1	0.65 ± 0.03	2.88E-15	3.51	12.02 (6.39 - 22.10)	1119.18 (444.56 - 4150.98)	107.84 (57.33 - 198.21)	124.08 (49.29 - 460.20)
2W	42 (888)	0	1.12 ± 0.06	1.49E-04	2.01	5.83 (4.15 - 8.11)	80.79 (50.97 - 146.13)	52.29 (37.25 - 72.73)	8.95 (5.65 - 16.20)
34H	48 (995)	0	0.86 ± 0.04	1.27E-06	2.30	5.83 (3.81 - 8.92)	174.55 (94.94 - 381.33)	52.26 (34.17 - 79.99)	19.35 (10.53 - 42.28)
36H	47 (977)	0	1.18 ± 0.06	4.23E-15	3.59	2.43 (1.54 - 3.82)	29.41 (16.35 - 66.16)	21.82 (13.79 - 34.27)	3.26 (1.81 - 7.33)
A01	42 (878)	2	0.96 ± 0.05	3.42E-47	5.08	27.31 (12.90 - 61.26)	582.08 (206.22 - 3564.65)	40.46 (8.50 - 135.61)	10.36 (2.77 - 271.05)
A07	50 (1108)	2	0.73 ± 0.04	1.19E-33	5.73	4.36 (2.04 - 8.525)	243.82 (93.64 - 1111.77)	39.14 (18.29 - 76.45)	27.03 (10.38 - 123.26)
AHC	55 (1180)	0	0.87 ± 0.04	9.77E-12	2.89	17.17 (11.09 - 26.90)	504.69 (261.35 - 1200.82)	153.95 (99.49 - 241.25)	55.95 (28.98 - 133.13)
AHS	52 (1146)	1	0.95 ± 0.05	1.45E-05	2.06	16.17 (11.47 - 22.98)	360.81 (213.53 - 700.50)	145.04 (102.83 - 206.12)	40.00 (23.68 - 77.66)
B06	42 (895)	2	1.22 ± 0.07	3.69E-06	2.33	6.31 (4.41 - 9.00)	70.24 (43.55 - 131.28)	56.64 (39.57 - 80.71)	7.79 (4.83 - 14.55)
B08	46 (975)	2	0.76 ± 0.04	6.58E-12	3.18	13.17 (7.84 - 22.09)	646.86 (288.62 - 2033.24)	118.13 (70.31 - 198.17)	71.71 (31.99 - 225.42)
B19	50 (1101)	4	1.01 ± 0.05	2.38E-08	2.54	28.49 (19.99 - 40.75)	532.21 (312.98 - 1062.37)	255.50 (179.33 - 365.43)	59.00 (34.69 - 117.78)
C03	47 (1002)	4	0.86 ± 0.05	1.55E-12	3.23	20.35 (12.56 - 33.09)	615.28 (302.79 - 1626.70)	182.55 (112.69 - 296.82)	68.21 (33.57 - 180.35)
C11	55 (1150)	4	0.81 ± 0.04	6.36E-07	2.67	24.23 (16.74 - 35.31)	908.24 (495.22 - 1995.14)	381.57 (194.71 - 810.61)	246.09 (78.07 - 1701.98)

C13	35 (745)	4	0.94 ± 0.06	5.10E-07	2.55	39.19 (24.67 - 64.79)	893.21 (429.53 - 2524.03)	412.99 (242.41 - 733.25)	99.28 (44.52 - 331.79)
C15	48 (994)	4	0.84 ± 0.04	2.98E-08	2.57	8.52 (5.44 - 13.32)	281.86 (145.09 - 685.04)	76.41 (48.83 - 119.44)	31.25 (16.08 - 75.95)
C18	59 (1250)	4	0.66 ± 0.03	2.98E-09	2.49	51.55 (32.46 - 84.55)	4509.64 (1951.23 - 13881.14)	462.27 (291.10 - 758.25)	499.96 (216.32 - 1538.94)
C21	51 (1075)	3	0.66 ± 0.04	1.21E-07	2.41	62.34 (38.30 - 105.58)	5483.84 (2242.88 - 18786.96)	559.05 (343.49 - 946.84)	607.97 (248.65 - 2082.82)
C24	52 (1090)	4	0.91 ± 0.05	2.14E-25	4.65	17.50 (10.14 - 29.83)	444.75 (208.26 - 1353.66)	156.94 (90.96 - 267.52)	49.31 (23.09 - 150.07)
D02	47 (979)	0	1.12 ± 0.06	1.44E-06	2.31	7.83 (5.48 - 11.22)	109.02 (65.86 - 209.96)	70.24 (49.13 - 100.63)	12.09 (7.30 - 23.28)
DPN	47 (976)	1	0.71 ± 0.04	2.00E-09	2.78	14.65 (8.62 - 24.35)	929.40 (426.20 - 2729.25)	131.43 (77.29 - 218.42)	103.04 (47.25 - 302.58)
PKR	48 (1000)	1	0.64 ± 0.04	5.32E-11	2.98	7.30 (3.92 - 12.81)	700.81 (298.73 - 2357.39)	65.49 (35.14 - 114.89)	77.69 (33.12 - 261.35)
WHE	47 (1000)	0	0.74 ± 0.04	2.31E-08	2.61	6.87 (4.06 - 11.07)	363.26 (183.83 - 909.05)	61.58 (36.42 - 99.27)	40.27 (20.38 - 100.78)

[a]Number of replicates (number of mosquitoes)
[b]Number of pyriproxyfen applications to catch basins in area
[c]P-value for Pearson's χ^2 goodness-of-fit test
[d]LC doses in ppb (μg/L)
