# Supplementary material for: Resistance to S-Methoprene Correlates with Pyriproxyfen Resistance in Field-Collected Culex pipiens
Source: Insects. 2026 Feb 26;17(3):241. doi: 10.3390/insects17030241 (PMC13027244; doi:10.3390/insects17030241)
Supplement: Supplementary file 1 [file insects-17-00241-s001.zip › Supplementary Table S7.pdf]

Supplementary Table S7. Summary of pyriproxyfen dose response data, resistance ratios, and statistical outputs for field-collected *Cx. pipiens*.

Collection Site	N(n)[a]	N PPF[b]	Slope ± s.e.m.	P-value[c]	H (χ^2/df)	LC_{50}[d] (95% CI)	LC_{90}[d] (95% CI)	RR_{50} (95% CI)	RR_{90} (95% CI)
COL	70 (1530)	0	0.51 ± 0.02	6.18E-46	2.25	0.003 (<0.001 - 0.01)	1.04 (0.29 - 6.93)	-	-
12P	54 (1162)	0	0.72 ± 0.04	9.09E-29	4.92	0.01 (<0.01 - 0.03)	0.96 (0.39 - 3.37)	5.42 (2.58 - 10.53)	0.92 (0.38 - 3.25)
15M	57 (1205)	1	0.63 ± 0.03	1.01E-21	4.01	0.07 (0.04 - 0.13)	7.78 (2.98 - 29.39)	23.01 (11.86 - 44.11)	7.49 (2.87 - 28.32)
17W	52 (1100)	0	0.52 ± 0.03	1.28E-20	4.09	0.04 (0.02 - 0.09)	13.26 (4.11 - 76.06)	15.21 (6.43 - 32.94)	12.78 (3.96 - 73.31)
21P	45 (956)	0	0.60 ± 0.04	5.84E-48	7.92	0.02 (<0.01 - 0.06)	2.91 (0.76 - 30.95)	7.36 (1.92 - 20.98)	2.81 (0.73 - 29.83)
23H	39 (815)	0	0.64 ± 0.04	1.79E-20	4.83	0.03 (0.01 - 0.06)	2.56 (0.74 - 20.68)	8.32 (3.53 - 18.94)	2.46 (0.71 - 19.94)
24S	42 (912)	0	0.53 ± 0.04	3.43E-26	5.42	0.02 (<0.01 - 0.05)	4.72 (1.16 - 53.45)	6.17 (1.95 - 16.02)	4.54 (1.12 - 51.52)
27S	47 (960)	1	0.69 ± 0.04	8.67E-13	3.27	0.01 (<0.01 - 0.01)	0.53 (0.23 - 1.66)	2.56 (1.25 - 4.77)	0.51 (0.11 - 1.60)
27W	42 (902)	0	0.59 ± 0.04	1.24E-26	3.64	0.15 (0.06 - 0.39)	22.38 (5.39 - 266.77)	38.78 (8.95 - 230.88)	67.31 (6.02 - 1248.44)
28E	36 (737)	1	0.64 ± 0.04	8.27E-26	5.94	0.01 (<0.01 - 0.03)	1.23 (0.34 - 12.78)	4.16 (1.25 - 10.82)	1.18 (0.32 - 12.32)
29M	54 (1170)	1	0.72 ± 0.03	6.78E-13	3.07	0.10 (0.06 - 0.17)	6.15 (2.87 - 16.86)	34.48 (20.40 - 58.03)	5.93 (2.77 - 16.25)
2W	48 (1024)	0	0.64 ± 0.04	1.32E-25	4.91	0.01 (<0.01 - 0.02)	0.90 (0.34 - 4.00)	2.95 (1.02 - 6.61)	0.86 (0.32 - 3.85)
34H	49 (1027)	0	0.68 ± 0.04	8.29E-13	3.21	0.01 (<0.01 - 0.02)	0.82 (0.36 - 2.51)	3.56 (1.89 - 6.33)	0.79 (0.34 - 2.42)
36H	48 (1008)	0	0.64 ± 0.04	3.24E-19	4.10	0.015 (<0.01 - 0.01)	0.57 (0.22 - 2.23)	1.91 (0.82 - 3.87)	0.55 (0.21 - 2.15)
A01	45 (935)	2	0.66 ± 0.04	1.21E-52	3.59	0.40 (0.14 - 2.06)	35.52 (5.22 - 2104.23)	30.17 (7.31 - 200.22)	17.03 (1.72 - 7103.40)
A07	46 (992)	2	0.64 ± 0.04	2.84E-31	5.76	0.04 (0.01 - 0.08)	3.64 (1.07 - 26.46)	11.64 (4.92 - 26.96)	3.51 (1.03 - 25.51)
AHC	54 (1137)	0	0.62 ± 0.03	2.62E-24	4.43	0.05 (0.02 - 0.10)	5.64 (2.05 - 23.48)	16.75 (8.02 - 34.04)	5.44 (1.98 - 22.64)
AHS	55 (1186)	1	0.52 ± 0.03	8.92E-21	3.98	0.02 (0.01 - 0.04)	5.85 (1.98 - 28.28)	7.04 (3.01 - 14.79)	5.64 (1.91 - 27.26)
B06	45 (970)	2	0.59 ± 0.03	9.38E-74	10.99	0.03 (0.01 - 0.09)	3.89 (0.77 - 98.29)	9.31 (2.26 - 31.64)	3.74 (0.74 - 94.74)
B08	50 (1077)	2	0.63 ± 0.03	1.65E-37	6.17	0.04 (0.01 - 0.09)	4.58 (1.34 - 32.56)	13.89 (5.79 - 32.69)	4.42 (1.28 - 31.38)
B19	61 (1342)	4	0.68 ± 0.03	9.65E-24	4.07	0.06 (0.03 - 0.10)	4.42 (1.85 - 14.59)	19.41 (10.91 - 34.58)	4.27 (1.79 - 14.07)
C03	54 (1157)	4	0.62 ± 0.03	3.81E-46	6.76	0.09 (0.04 - 0.22)	10.45 (2.90 - 81.58)	30.43 (12.78 - 73.49)	10.08 (2.79 - 78.63)

C11	57 (1217)	4	0.54 ± 0.03	3.54E-35	4.11	0.19 (0.08 - 0.45)	42.39 (10.41 - 422.83)	274.82 (91.49 - 1354.66)	126.41 (16.27 - 11587.7)
C13	41 (876)	4	0.78 ± 0.05	1.58E-30	5.86	0.43 (0.19 - 1.01)	19.16 (6.00 - 139.17)	282.69 (123.81 - 704.41)	22.25 (6.91 - 189.44)
C15	57 (1220)	4	0.79 ± 0.04	2.19E-36	5.56	0.09 (0.04 - 0.17)	3.78 (1.62 - 12.69)	30.22 (14.95 - 57.43)	3.64 (1.56 - 12.24)
C18	74 (1634)	4	0.79 ± 0.03	1.56E-36	4.73	0.46 (0.27 - 0.79)	19.28 (8.88 - 54.58)	152.94 (89.45 - 263.89)	18.58 (8.56 - 52.61)
C21	60 (1301)	3	0.63 ± 0.03	6.36E-19	3.60	0.14 (0.08 - 0.26)	15.28 (6.34 - 50.59)	48.31 (26.84 - 86.02)	14.73 (6.11 - 48.76)
C24	58 (1277)	4	0.72 ± 0.03	3.73E-37	5.57	0.09 (0.04 - 0.18)	5.33 (2.24 - 18.25)	30.06 (14.02 - 58.77)	5.14 (2.16 - 17.59)
D02	51 (1081)	0	0.62 ± 0.03	4.24E-30	5.25	0.05 (0.02 - 0.11)	5.58 (1.75 - 32.28)	15.99 (7.17 - 35.53)	5.38 (1.68 - 31.12)
DPN	50 (1034)	1	0.86 ± 0.04	7.92E-06	2.13	0.09 (0.06 - 0.15)	2.98 (1.69 - 6.08)	32.43 (21.03 - 49.01)	2.87 (1.64 - 5.86)
PKR	52 (1125)	1	0.82 ± 0.04	1.70E-07	2.37	0.16 (0.10 - 0.24)	5.69 (3.13 - 12.19)	51.89 (33.14 - 79.83)	5.49 (3.02 - 11.75)
WHE	51 (1062)	0	0.69 ± 0.04	2.45E-08	2.52	0.05 (0.03 - 0.08)	3.58 (1.79 - 8.73)	17.22 (9.86 - 28.86)	3.45 (1.73 - 8.41)

[a]Number of replicates (number of mosquitoes)

[b]Number of pyriproxyfen applications to catch basins in area

[c]P-value for Pearson's χ^2 goodness-of-fit test

[d]LC doses in ppb (μg/L)
