# Supplementary material for: Resistance to S-Methoprene Correlates with Pyriproxyfen Resistance in Field-Collected Culex pipiens
Source: Insects. 2026 Feb 26;17(3):241. doi: 10.3390/insects17030241 (PMC13027244; doi:10.3390/insects17030241)
Supplement: Supplementary file 1 [file insects-17-00241-s001.zip › Supplementary Table S8.pdf]

Supplementary Table S8. Model selection table. Models within 2 AIC were considered equal. Final model selection is bold. Reporting includes the terms included in the regression, the distribution (negative binomial), df (K), AIC, Delta AIC, AIC weight, and Log Likelihood.

Model	Regression terms	PDF	K	AICc	Delta AIC	AICcWt	LL
**PPF RR_{50}**	**S-methoprene RR_{50} + Years PPF use**	**nb**	**4**	**261.19**	**0**	**0.62**	**-125.82**
	S-methoprene RR_{50}	nb	3	262.35	1.04	0.36	-127.67
	Years PPF use	nb	3	270.33	9.14	0.02	-131.72
**PPF RR_{90}**	**S-methoprene RR_{90} + Years PPF use**	**nb**	**4**	**269.45**	**0**	**0.61**	**-129.95**
	Years PPF use	nb	3	270.33	0.88	0.39	-131.72
	S-methoprene RR_{90}	nb	3	281.63	12.18	0.00	-137.37
